# Diagnostic accuracy and clinical usefulness of erythrocyte creatine content to predict the improvement of anaemia in patients receiving maintenance haemodialysis

**DOI:** 10.1186/s12882-022-03055-4

**Published:** 2023-01-03

**Authors:** Ohki Hayashi, Seishi Nakamura, Tetsuro Sugiura, Shun Hasegawa, Yoshiaki Tsuka, Nobuyuki Takahashi, Sanae Kikuchi, Koichiro Matsumura, Toshika Okumiya, Masato Baden, Ichiro Shiojima

**Affiliations:** 1 Department of Nephrology and Cardiology, Takarazuka Hospital, 2-1-2 Nogami, Takarazuka, 665-0022 Hyogo, Japan; 2 Department of Internal Medicine II, Kansai Medical University, Osaka, Japan; 3 Department of Nephrology, Kansai Medical University Kori Hospital, Osaka, Japan; 4 Cardiovascular Medicine, Faculty of Medicine, Kindai University, Osaka, Japan; 5 Department of Medical Laboratory Science, Kochi Gakuen University, Kochi, Japan

**Keywords:** Erythrocyte creatine content, Erythropoiesis stimulating agent, Erythropoietin resistance, Haemodialysis, Renal anaemia

## Abstract

**Background:**

The improvement of anaemia over time by erythropoiesis stimulating agent (ESA) is associated with better survival in haemodialysis patients. We previously reported that erythrocyte creatine content, a marker of erythropoietic capacity, was a reliable marker to estimate the effectiveness of ESA. The aim of this study was to examine the accuracy and clinical usefulness of erythrocyte creatine content to predict the improvement of anaemia in haemodialysis patients.

**Methods:**

ESA dose was fixed 3 months prior to the enrollment and was maintained throughout the study period. Erythrocyte creatine content and haematologic indices were measured at baseline in 92 patients receiving maintenance haemodialysis. Haemoglobin was also measured 3 months after. Improvement of anaemia was defined as ≥ 0.8 g/dL change in haemoglobin from baseline to 3 months.

**Results:**

Erythrocyte creatine content was significantly higher in 32 patients with improvement of anaemia compared to 60 patients with no improvement of anaemia (2.47 ± 0.74 vs. 1.57 ± 0.49 μmol/gHb, *P* = 0.0001). When 9 variables (erythrocyte creatine content, ESA dose, reticulocyte, haptoglobin, haemoglobin at baseline, serum calcium, intact parathyroid hormone, transferrin saturation and serum ferritin) were used in the multivariate logistic regression analysis, erythrocyte creatine emerged as the most important variable associated with the improvement of anaemia (*P* = 0.0001). The optimal cut-off point of erythrocyte creatine content to detect the improvement of anaemia was 1.78 μmol/gHb (Area under the curve: 0.86). Sensitivity and specificity of erythrocyte creatine content to detect the improvement of anaemia were 90.6% and 83.3%.

**Conclusion:**

Erythrocyte creatine content is a reliable marker to predict the improvement of anaemia 3 months ahead in patients receiving maintenance haemodialysis.

## Background

Anaemia, mainly due to insufficient production of erythropoietin, is a common complication in patients with end-stage renal disease and is associated with poor long-term prognosis [1]. Erythropoiesis stimulating agent (ESA), a potent haematopoietic growth factor, is used to predict the improvement of anaemia in patients receiving maintenance haemodialysis [2–5]. Most studies have examined the association between baseline haemoglobin value and subsequent survival [6–8], but haemoglobin may change drastically over time due to the changes in ESA dose and/or intravenous iron dose. Therefore, longitudinal measurement of haemoglobin rather than one point is reported to provide more accurate information in patients receiving maintenance haemodialysis [9]. Young erythrocytes contain substantially higher creatine levels than older erythrocytes and creatine contents in erythrocytes decrease gradually with advancing cell age [10–12]. Accelerated red cell production, which lead to an increase in the young erythrocytes results in red cells containing higher erythrocyte creatine levels and hence, erythrocyte creatine level reflects average or cumulative erythropoiesis [13–19]. In patients with normal erythropoietic capacity such as intravascular haemolysis and haemolytic anaemia, red cell production is accelerated in proportion to the amount of erythrocyte destruction, which lead to an increase in the young erythrocytes with higher erythrocyte creatine levels [13–17]. In contrast, renal anaemia is mainly caused by decreased erythropoietic capacity due to inadequate erythropoietin production [20–25]. Therefore, the erythrocyte creatine content is regarded as an index of erythropoiesis rather than average erythrocyte age in haemodialysis patients receiving ESA treatment with renal anaemia [17–19]. We previously reported that erythrocyte creatine content, a marker of erythropoietic capacity, is reliable to estimate the effectiveness of ESA in haemodialysis patients [26]. However, optimal cut-off point of erythrocyte creatine content to predict the improvement of anaemia was not established. Accordingly, the aim of this study was to derermine diagnostic accuracy and clinical usefulness of erythrocyte creatine content to predict the improvement of anaemia in patients receiving maintenace haemodialysis.

## Methods

### Study patients

We assessed patients aged ≥ 20 years who had been receiving maintenance haemodialysis 3 times a week for at least 6 months in the Dialysis Unit at Takarazuka Hospital or at Kansai Medical University Hospital. All of our outpatients on haemodialysis were maintaining quality of life and took ordinary dialysis-diet with no extra creatine supplementation. None of our patients were vegetarians. The exclusion criteria were as follows: bleeding event within 3 months, infection requiring parenteral antibiotics, mechanical heart valves, concurrent malignancy, haemolytic disease or blood transfusion within 3 months.

### Study protocol

**Fig. 1:**
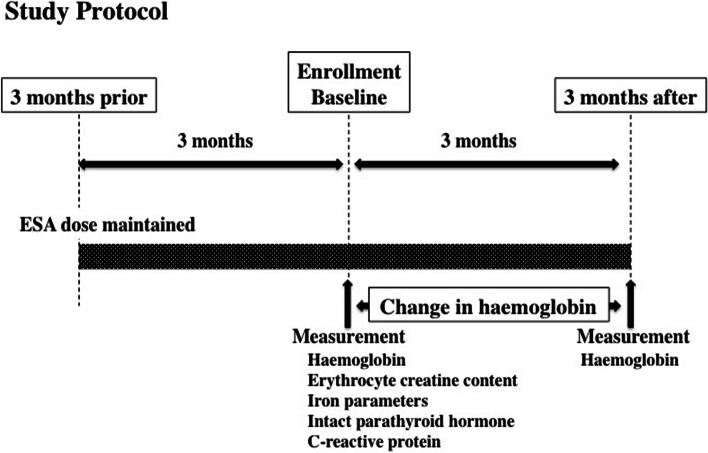
Study protocol The dose of erythropoiesis stimulating agent (ESA) was fixed 3 months prior to the enrollment and was maintained throughout 6 months period. Laboratory tests were performed at baseline and haemoglobin was measured 3 months later

Study protocol is shown in Fig. 1. ESA treatment was fixed for a duration of 3 months prior to the enrollment and was maintained throughout the study period. Baseline blood examination including erythrocyte creatine content and other laboratory examination (haemoglobin, reticulocyte, haptoglobin, transferrin saturation, ferritin, intact parathyroid hormone, serum calcium, serum phosphorus, serum albumin and C-reactive protein) were performed. Intravenous iron treatment with 40 mg ferric saccharate 3 times a week was administered at the end of each haemodialysis session and was followed by 100 mg of oral iron supplements in patients with absolute iron deficiency, defined as transferrin saturation < 20% and serum ferritin < 100 ng/ml [27]. Anaemia was defined as haemoglobin < 10 g/dL at baseline according to the Guidelines of Japanese Society for Dialysis therapy [27, 28] and improvement of anaemia was defined as ≥ 0.8 g/dL change in haemoglobin from baseline to 3 months [9]. Patients were divided into 2 groups; Group 1: ≥ 0.8 g/dL change in haemoglobin from baseline to 3 months and Group 2: < 0.8 g/dL change in haemoglobin. This study protocol was reviewed and approved by Takarazuka Hospital ethical committees for human research, approval number (No. 2022002). All the patients provided written informed consent and the investigation conformed to the principles outlined in the Declaration of Helsinki.

### Haemodialysis

All patients were dialysed for 4.0–8.0 h, using a single-use dialyser: cellulose FB, Nipro Corporation, Osaka, Japan), poly-sulfone, (PN, Nikkiso Co., Ltd., Tokyo, Japan), polyethersulfone (PES, Nipro Corporation, Osaka, Japan), or polymethylmethacrylate (NF-H, Toray Medical Co., Ltd., Tokyo, Japan) with a 1.3–2.1 m^2^ effective surface area. All patients received haemodialysis with blood flow of 200 ml/min with dialysate flow of 500 ml/min. In all patients, haemodialysis was performed via native arteriovenous fistulas with a dual plastic needle and 16-gauge cannula. The patients uniformly received a dialysate (D-dry, Nikkiso Co., Ltd., Tokyo, Japan) and an anticoagulant with heparin sodium. Bolus of heparin sodium 1,000 units was intravenously administrated at the start of haemodialysis followed by continuous administration of 500 to 750 units/hour. The dialysate temperature of extracorporeal circulation was strictly maintained at 36–38 °C [11, 12, 19]. ESA and iron therapy were prescribed in accordance with the Guidelines of Japanese Society for Dialysis therapy [27, 28]. ESA therapy with epoetin beta pegol or darbepoetin alfa was administrated at the end of haemodialysis. Haemodialysis time (hours/week), intradialytic ultrafiltration rates (ml/hour/kg) were measured and Kt/V, as an index of urea clearance, was calculated. These indices were calculated as the average of 3 consecutive haemodialysis sessions. One of the following dialysis membranes was used: cellulose FB (Nipro Corporation, Osaka, Japan), poly-sulfone (PN, Nikkiso Co., Ltd., Tokyo, Japan), polyethersulfone (PES, Nipro Corporation, Osaka, Japan), or polymethylmethacrylate (NF-H, Toray Medical Co., Ltd., Tokyo, Japan) [17, 26].

### Laboratory measurements

Blood samples were drawn immediately before the haemodialysis. Haematologic examinations and reticulocyte count were carried out with a Sysmex XN 1000 (Sysmex, Kobe, Japan). Haptoglobin was measured by the TIA method with JCA-BM 6010 (JEOL, Tokyo, Japan). Serum iron was measured by the Nitroso-PSAP method with AU 5840 (Beckman Coulter; Tokyo, Japan), unsaturated iron binding capacity (UIBC) by the Nitroso-PSAP method with BM-6050 (JEOL, Tokyo, Japan) and ferritin by radioimmunoassay with AU-5840 (Beckman Coulter, Tokyo, Japan). Transferrin saturati4on was calculated as: [Serum iron/ (Serum iron + UIBC)] × 100 (%). Intact parathyroid hormone was measured by the ECLIA method with Cobas 8000 (Roche Diagnostics, Tokyo, Japan). Other biochemical laboratory measurements were performed by TBA-120 FR automated biochemical analyzer (Canon, Osaka, Japan). A weekly ESA dose was administered as a darbepoetin alfa equivalent dose. ESA was converted using the following formula: darbepoetin alfa (μg) = epoetin beta pegol (μg) × 0.8 = epoetin (U) × 200, in accordance with previous reports [17–26]. Post-haemodialysis weight was measured as a body weight. ESA cut-off point of high and low dose of ESA was defined as 9,000 units/ week in this study according to Tsubakihara et al. [28]

### Measurement of the erythrocyte creatine content

Erythrocyte creatine content levels were assayed using highly sensitive enzymatic method in accordance with previous reports [11, 12, 29]. Briefly, blood samples were collected in ethylenediaminetetraacetic acid-containing tubes and centrifuged to remove the plasma and buffy coat. After lysis and deproteinisation of packed erythrocytes, the supernatant was obtained by centrifugation and filtration. The erythrocyte creatine content assay had an excellent intra (with-run CVs < 1.0%) and inter (between-day CVs < 2.0%) assay variation [29]. Measured data are expressed as micromole per gram of haemoglobin (μmol/gHb).

### Statistical analyses

Results are expressed as mean ± standard deviation. Statistical analyses between the 2 groups were performed by one-way layout analysis of variance or chi-square analysis followed by Scheffe type multiple comparison method. The multivariate logistic regression analysis was performed to evaluate the important variables related to the improvement of anaemia. The optimal cut-off point of erythrocyte creatine content to detect patients with the improvement of anaemia was calculated by the receiver-operating characteristic analysis. A probability value of < 0.05 was considered significant. Parameters were compared with the use of commercially available statistical software (StatView, Abacus Concepts, Berkeley, CA). Multivariate logistic regression analysis and receiver-operating characteristic analysis was performed using the JMP 14. 2.0 software (SAS Institute Inc., Cary, NC, USA).

## Results

A total of 92 outpatients (62 men and 30 women, mean age of 72 ± 14 years) were included in the study. Patients with bleeding event within 3 months (*n* = 3), infection requiring parenteral antibiotics (*n* = 2) and mechanical heart valves (*n* = 3) were excluded. None of the patients had concurrent malignancy, haemolytic disease or blood transfusion within 3 months. Clinical characteristics are shown in Table 1. There were no significant differences in clinical characteristics, haemodialysis condition and laboratory measurements (reticulocyte, haptoglobin, transferrin saturation, ferritin, incidence of iron deficiency, intact parathyroid hormone, C-reactive protein, serum calcium, serum phosphorus and albumin) between the two groups, but erythrocyte creatine content was significantly higher in Group 1 compared to Group 2 (2.47 ± 0.74 versus 1.57 ± 0.49 μmol/gHb, *P* = 0.0001). Haemoglobin at baseline was significantly lower in Group 1 compared to Group 2 (10.0 ± 1.3 versus 10.6 ± 1.0, g/dL *p* = 0.004), but haemoglobin at 3 months was significantly higher in Group 1 compared to Group 2 (11.1 ± 1.4 versus 10.3 ± 1.2 g/dL, *p* = 0.007). Nineteen (59.3%) patients in Group 1 received ≥ 9,000 units/week of ESA, whereas 40 (66.7%) patients in Group 2 received < 9,000 units/week of ESA (Chi-square = 5.80, *P* = 0.016). To determine the important variables related to the improvement of anaemia, 9 variables (erythrocyte creatine content, ESA dose, reticulocyte, haptoglobin, haemoglobin at baseline, serum calcium, intact parathyroid hormone, transferrin saturation and serum ferritin) were used in the multivariate logistic regression analysis. As a result, erythrocyte creatine content was the most important variable related to the improvement of anaemia (Chi-square = 18.72 , *P* = 0. 0001; Table 2).

**Table 1:** Clinical characteristics

	Study Patients	^a^Group 1	^b^Group 2	*P* Value
	(*n* = 92)	(*n* = 32)	(*n* = 60)	
Age (years)	71.9 ± 14.0	73.7 ± 12.9	71.0 ± 14.5	0.37
Gender (male/female)	62/30	20/12	42/18	0.49
Diabetes Mellitus (resent/absent)	42/50	16 / 16	26 / 34	0.54
Haemodialysis time (hours/week)	15.9 ± 4.2	15.3 ± 3.3	16.2 ± 4.6	0.36
Intradialytic ultrafiltration rate (ml/hour/kg)	8.8 ± 2.9	9.1 ± 3.0	8.6 ± 2.9	0.40
Kt/v	1.72 ± 0.48	1.7 ± 0.5	1.8 ± 0.5	0.38
Erythrocyte creatine content (μmol/gHb)	1.89 ± 0.73	2.47 ± 0.74	1.57 ± 0.49	0.0001
ESA dose (units/week)	6,396.7 ± 3,746.3	7429.7 ± 3623.0	5845.8 ± 3723.2	0.04
Low dose (< 9,000)	53	13	40	0.016
High dose (≥ 9,000)	39	19	20	
Haemoglobin at baseline (g/dL)	10.4 ± 1.2	10.0 ± 1.3	10.6 ± 1.0	0.004
Haemoglobin at 3 months (g/dL)	10.6 ± 1.3	11.1 ± 1.4	10.3 ± 1.2	0.007
Reticulocyte (%)	14.1 ± 6.2	14.3 ± 7.3	14.0 ± 5.6	0.81
Haptoglobin (g/dL)	93.6 ± 52.2	101.1 ± 52.5	89.7 ± 52.0	0.32
Transferrin saturation (%)	27.8 ± 16.2	23.5 ± 12.3	28.5 ± 17.4	0.16
Ferritin (ng/mL)	121.3 ± 106.5	100.6 ± 89.4	145.6 ± 245.3	0.32
Iron deficiency (present/ absent)	38/54	16/16(50.0%)	22/38(36.7%)	0.27
Intact parathyroid hormone (pg/mL)	152.3 ± 102.0	170.3 ± 139.5	143.7 ± 77.9	0.30
C-reactive protein (mg/dL)	0.33 ± 0.37	0.38 ± 0.46	0.30 ± 0.31	0.33
Serum calcium (mg/dL)	9.5 ± 0.5	9.4 ± 0.5	9.5 ± 0.5	0.25
Serum phosphorus (mg/dL)	4.9 ± 1.4	4.7 ± 1.4	5.0 ± 1.3	0.32
Albumin (g/dL)	3.6 ± 0.4	3.5 ± 0.4	3.7 ± 0.4	0.05

Data are represented as mean ± SD. *ESA* erythropoiesis stimulating agent, *Kt/v* urea clearance

^a^Group 1 ≥ 0.8 g/dL change in haemoglobin from baseline to 3 months

^b^Group 2 < 0.8 g/dL change in haemoglobin from baseline to 3 months

**Table 2:** Factors related to the ^a^improvement of anaemia

			95% Confidence Interval		*P* Value
	Odds ratio	Chi-square	Lower	Upper	
Erythrocyte creatine content (μmol/gHb)	**48.82**	**18.72**	**8.39**	**284.17**	**0.0001**
ESA dose (units/week)	**1.00**	**4.45**	**0.99**	**1.01**	**0.04**
Reticulocyte (%)	**1.05**	**0.63**	**0.93**	**1.18**	**0.43**
Haptoglobin (g/dL)	**1.01**	**4.63**	**1.00**	**1.03**	**0.031**
Haemoglobin at baseline (g/dL)	**0.41**	**5.73**	**0.19**	**0.85**	**0.017**
Serum calcium (mg/dL)	**0.98**	**0.001**	**0.22**	**4.24**	**0.97**
Intact parathyroid hormone (pg/mL)	**1.00**	**0.03**	**0.99**	**1.01**	**0.87**
Transferrin saturation (%)	**1.01**	**0.14**	**0.96**	**1.07**	**0.71**
Serum ferritin (ng/mL)	**1.00**	**0.02**	**0.99**	**1.01**	**0.98**

*ESA* erythropoiesis stimulating agent

^a^Improvement of anaemia ≥ 0.8 g/dL change in haemoglobin from baseline to 3 months

### Diagnostic accuracy

**Fig. 2:**
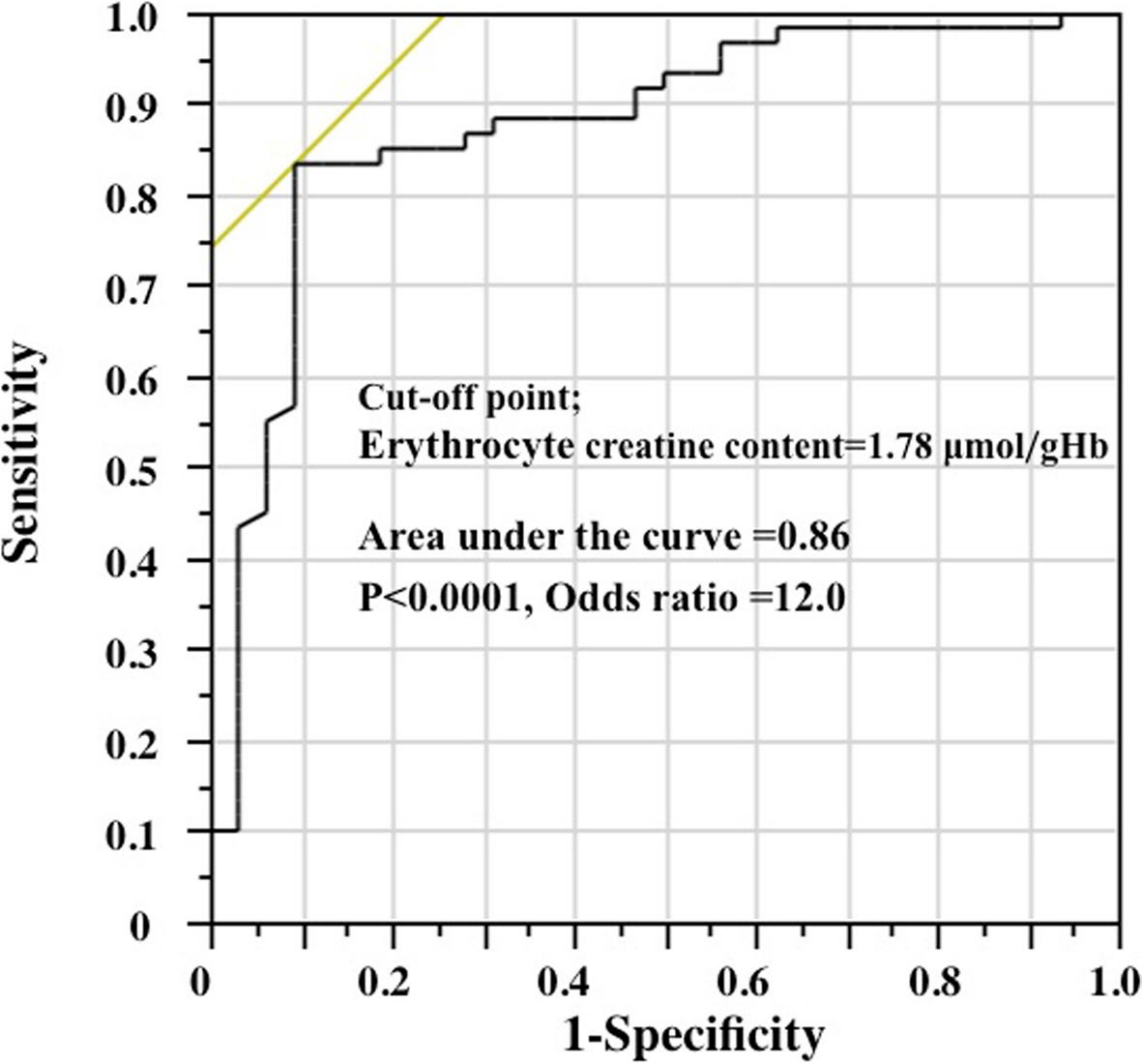
Receiver-operating characteristic curve to detect the improvement of anaemia. ESA, erythropoiesis stimulating agent Improvement of anaemia; ≥ 0.8 g/dL change in haemoglobin from baseline to 3 months.

The optimal cut-off point of erythrocyte creatine content to detect the improvement of anaemia was 1.78 μmol/gHb by the receiver operating characteristic curve; high area under the curve (0.86) and good predictability (Fig. 2). Sensitivity, specificity, positive predictive value and negative predictive value of erythrocyte creatine content to detect patients with the improvement of anaemia were 90.6%, 83.3%, 74.4% and 94.3%, respectively. Among 39 patients with erythrocyte creatine content > 1.78 μmol/gHb, 29 (74.3%) patients showed increase in haemoglobin, whereas haemoglobin did not increase in 50 (94.3%) of 53 patients with erythrocyte creatine content ≤ 1.78 μmol/gHb; difference significant (Chi-square = 46.7, *P* < 0.0001; Table 3). Twenty-five (64.1%) of 39 patients with erythrocyte creatine content > 1.78 μmol/gHb were receiving high dose ESA (≥ 9,000 units/week) and 14 patients low dose ESA (< 9,000 units/week), whereas 39 (73.6%) of 53 patients with erythrocyte creatine content ≤ 1.78 were receiving low dose ESA and high dose ESA in 14 patients (Chi-square = 13.1, *P* < 0.0003; Table 4).

**Table 3:** Diagnostic accuracy of erythrocyte creatine

		^a^Improvement of anaemia	
		Yes	No
Erythrocyte creatine content (μmol/gHb)	≤ 1.78	3	50
	> 1.78	29	10

Chi-square = 46.7, *P* < 0.0001

^a^Improvement of anaemia ≥ 0.8 g/dL change in haemoglobin from baseline to 3 months

**Table 4:** Erythrocyte creatine and dose of ESA

		Dose of ESA, units/week	
		< 9,000	≥ 9,000
Erythrocyte creatine content (μmol/gHb)	≤ 1.78	39	14
	> 1.78	14	25

Chi-square = 13.1, *P* < 0.0003

*ESA* erythropoiesis stimulating agent

## Discussion

Anaemia is a common complication in end-stage renal disease and is associated with poor long-term prognosis [21–24]. Renal anaemia is mainly due to decrease in erythropoietic capacity caused by inadequate erythropoietin production and/or resistance to ESA treatment [1, 24–26]. ESA accelerates erythropoiesis and correcting anaemia by ESA is related to the improvement of quality of life and leads to better prognosis [6–8]. However, most of the studies examined the effect of ESA by single haemoglobin or haematocrit level at the start of the study and did not assess the variation of haemoglobin over time [6–8, 21, 22]. In our previous report, we clarified that erythrocyte creatine content can assess accelerated erythropoietic capacity by ESA and is useful to estimate ameliorative effectiveness of ESA in haemodialysis patients [26]. However, optimal cut-off point of erythrocyte creatine content to predict the improvement of anaemia was not determined. Increase in hemoglobin was defined as haemoglobin change of 0.1 g/dL [26], but this cut-off value may be influenced by small variation in hydration status. In a previous study with 58,058 patients receiving maintenance haemodialysis, patients with ≥ 0.8 g/dL increase in haemoglobin over time had lower risk of death independent of baseline haemoglobin level [9]. In this study, we defined the improvement of anaemia as ≥ 0.8 g/dL increase in haemoglobin from baseline to 3 months in this study and investigated the diagnostic accuracy and clinical usefulness of erythrocyte creatine to predict the improvement of anaemia using the same protocol in a larger number of haemodialysis patients. Moreover, the optimal frequency of follow up testing undergoing ESA treatment in patients with anaemia is not known. The Kidney Disease: Improving Global Outcomes (KDIGO) guidelines recommended to measure haemoglobin at least every 3 months in patients with anaemia [30]. Beguin et al. reported that there were 3 groups of responders to ESA treatment; 34 early responders (respond within 3 months after baseline examination), 15 late responders (respond 3–6 months after baseline examination) and 15 non-responders [20]. This study emphasized the clinical importance of early recognition of erythropoietic response to adjust dose of ESA and to identify specific cause such as subclinical inflammation or iron deficiency and treatment. Therefore, we defined follow up period as 3 months, and found that erythrocyte creatine content was the most important marker predicting the improvement of anaemia. Moreover, a cut-off point of 1.78 μmol/gHb had an excellent diagnostic accuracy in predicting the improvement of anaemia. Thus, erythrocyte creatine content, an index of erythropoiesis, is a reliable marker predicting the improvement of anaemia 3 months ahead receiving ESA treatment.

ESA resistance, related to increased mortality, is defined as lack of improvement anaemia despite receiving high dose of ESA [21–25]. Previous studies demonstrated that a significant number of patients failed to improve anaemia despite modifying many factors to optimize patients’ response to ESA [9, 27, 28]. Several factors are involved in ESA resistance, such as iron deficiency, inflammation, hyperparathyroidism or bone marrow dysfunction [21, 24, 25]. In this study, there were no significant differences in haptoglobin, C-reactive protein, serum ferritin and intact parathyroid hormone between Group 1 and Group 2. Iron deficiency, one of the causes of ESA resistance, has been widely investigated [20, 27, 28, 31]. Because patients with absolute iron deficiency were treated by iron supplements, there was no significant differences in the incidence of iron deficiency at baseline, evaluated by transferrin saturation and serum ferritin Group 1 and Group 2. Therefire, iron deficiency was not a reason of ESA resistance in this study.

At least 1–2 months are needed to evaluate the effect of ESA on the improvement of anaemia [27, 30]. However, due to the long life-span of mature red blood cell, erythrocyte indices do not provide information of the rapid change in erythropoietic activity. In contrast, erythrocyte creatine content, a quantitative marker of mean red blood cell age, because young blood cells contain higher creatine levels than older blood cells, reflects average or cumulative erythropoiesis during 3 months of ESA treatment (between 3 months prior to the enrollment and the baseline measurement) [17–19]. As patients in Group 1 had significantly higher erythrocyte creatine content level and were receiving significantly higher dose of ESA. These data indicate that 32 patients in Group 1 were good responders to ESA due to acceleration of erythropoiesis by ESA judged by the higher erythrocyte creatine content. Lack of improvement of anemia observed in 40 of 60 patients (Group 2) was due to under-dosing of ESA (< 9,000 units/week). In constrast, ESA resistance may have existed in 20 of 60 patients receiving high dose of ESA (≥ 9,000 units/week) in Group 2.

### Clinical implications (Fig. 3)

**Fig. 3:**
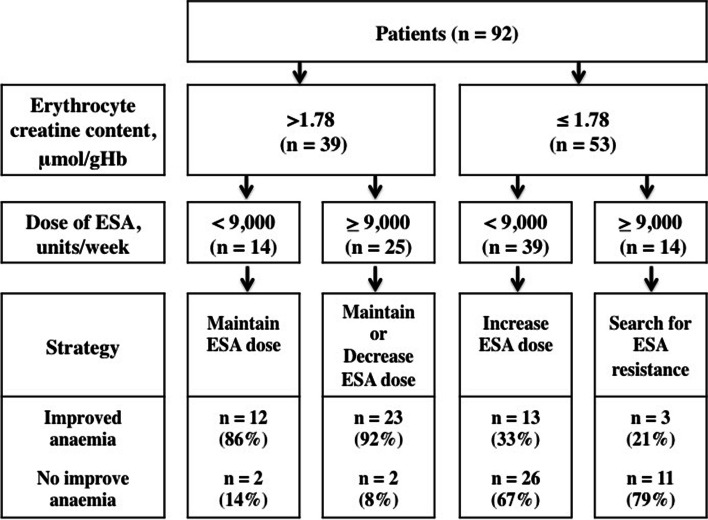
Treatment strategy. ESA, erythropoiesis stimulating agent

The dose of ESA is recommended to be maintained in patients achieving target haemoglobin with high erythrocyte creatine content value (> 1.78 μmol/gHb), whereas the dose of ESA should be reduced in patients exceeding target haemoglobin to avoid complication caused by excessive ESA. On the other hand, improvement of anaemia could be achieved by giving a higher dose of ESA in patients with low erythrocyte creatine content value (≤ 1.78 μmol/gHb) receiving < 9,000 units/week of ESA, whereas ESA resistance should be searched in patients with low erythrocyte creatine content value (≤ 1.78 μmol/gHb) despite receiving ≥ 9,000 units/week of ESA. Thus, measurement of erythrocyte creatine content provides a valuable information involving ESA therapeutic strategy in patients receiving maintenance haemodialysis and should therefore be included routinely for surveillance of dialysis patients for monitoring the therapeutic utiliy of ESA and to prevent the risk of anaemia.

### Limitations

Four limitations of this study should be addressed. First, this study is limited by a small number of patients. Further studies are warranted to confirm the prognostic significance of erythrocyte creatine content in a large group of haemodialysis patients receiving ESA. Second, we measured erythrocyte creatine content levels after keeping the same dose of ESA 3 months before the baseline measurement. However, potential wash-out effect could have remained during this 3 months. Nonetheless, erythrocyte creatine content, not reticulocyte count, emerged as an important variable of erythropoiesis. Third, the change in haemoglobin is influenced by iron status [20, 28, 31]. Reticulocyte haemoglobin content and/or high-fluorescence reticulocyte count are reported to be better predictors of iron-deficient erythropoiesis in patients on ESA treatment [31, 32]. Although we did not measure reticulocyte haemoglobin content or high-fluorescence reticulocyte count, intravenous and oral iron supplements were given as needed to avoid possible functional iron deficiency. Fourth, although non of our patients were taking creatine creatine supplements, creatine supplementation is recommended to maintain endogenous creatine pools in haemodialysis patients with impaired creatine homeostasis [33, 34]. Basal erythrocyte creatine content of vegetarians reported to be significantly lower than that of meat-eaters [35]. Further study is needed to investigate clinical significance of erythrocyte creatine content in patients taking creatine supplementation or vegetarians, but erythrocyte creatine content is an accurate indicator of erythropoietic activity among patients with ordinary dialysis-diet, who are not taking creatine supplementation.

## Conclusion

By a single blood sample measurement, erythrocyte creatine content can accurately predict the improvement of anaemia 3 months ahead in patients receiving maintenance haemodialysis.

## Data Availability

The datasets used and/or analysed during the current study are available from the corresponding author on reasonable request.

## Abbreviations

ESA: erythropoiesis stimulating agent
